# An introduction to the civil registration and vital statistics systems with applications in low- and middle-income countries

**DOI:** 10.1186/s41043-019-0177-1

**Published:** 2019-10-18

**Authors:** Samuel Mills, Jane Kim Lee, Bahie Mary Rassekh

**Affiliations:** World Bank Group, 1818 H Street, NW, Washington, DC, 20433 USA

**Keywords:** Civil registration, Birth registration, Vital statistics, Unique identification number

## Abstract

In collaboration with development partners, the World Bank Group (WBG) has been working to strengthen civil registration and vital statistics (CRVS) systems in low- and middle-income countries through lending operations, technical assistance projects, advisory services and analytics, and knowledge sharing at various international, regional, and national conferences and fora and through publications. In 2017, it launched a comprehensive CRVS eLearning course, which provides practical tools and approaches to achieving twenty-first-century state-of-the-art CRVS systems that are linked to identity management systems and are tailored to local contexts. Some of the key lessons learned from the various initiatives and projects are presented in the eight peer-reviewed manuscripts included in this issue.

## Main text

### What is civil registration and vital statistics (CRVS)?

Civil registration is the *universal*, *continuous*, *permanent*, *and compulsory recording of vital events occurring in a country’s population according to the legal requirements of each country* [1]. There are ten vital events that are recommended by the United Nations for recording: live birth, death, fetal death, marriage, divorce, annulment of marriage, judicial separation of marriage, adoption, legitimation, and recognition [2]. Vital statistics *constitute the collection of statistics on vital events pertaining to the population*, *including relevant characteristics of the events themselves and also of the persons concerned* [3] and are obtained from the civil registration system. Other sources of vital statistics are population surveys and censuses.

### Why improve the CRVS systems in low- and middle-income countries (LMICs)?

CRVS is a fundamental responsibility of the national government, and its primary functions can be largely categorized into three aspects: legal, administrative, and statistical [4]. First and foremost, civil registration establishes an individual’s legal identity. In some countries, legal documents (e.g., a birth certificate) are often needed by individuals for proof of name and other facts surrounding their identity when applying for access to government services such as (i) access to education, health, age-based cash transfer, and other government social services; (ii) access to formal employment and benefits, for example, death and disability insurance as well as health insurance; and (iii) the right to claim inheritance, among others.

Second, the CRVS system plays an important role in supporting the administrative functions of the government. Based on the data that are recorded through civil registration, the government can have access to reliable and continuous population data to be able to make informed decisions on government policies, programs, and services. Disaggregated data from civil registration can inform the condition and needs of the population at any size and administrative-level to enable the government to carry out more targeted planning and monitoring of programs, resulting in improved resource allocation. Data from the CRVS system are also fed into other government and functional registers, for example, the national identity management system, the population register, the electoral roll, and cancer registries, to support various public and private sectors in using the data to carry out their services.

Vital statistics compiled from complete and accurate civil registration data provide the optimal source of data for monitoring both national and subnational level development plans as well as for monitoring the Sustainable Development Goals (SDGs). By definition, CRVS can provide data at the national and lower administrative levels on a continuous basis. This is not the case for population censuses that are conducted approximately every 10 years, or household surveys that are often conducted every 3 to 5 years. Household surveys also do not provide data at the lower administrative levels, and their estimates have sampling errors. Well-functioning CRVS systems are the best source of data for monitoring 12 of the 17 SDGs, and 67 of the 232 SDG indicators [5]. Yet, more than 110 LMICs do not have functioning CRVS systems [6].

As a response to this need, the *Global CRVS Scaling Up Investment Plan 2015–2024* [6] was published in May 2014 to bring together global collective efforts for improving CRVS systems in LMICs. The WBG, which led the development of the investment plan, subsequently prepared the *WBG 2016–2030 CRVS Action Plan* which was endorsed by the WBG Data Council in December 2015 [7]. The overall objective of the Action Plan is “achieving universal civil registration of births, deaths, and other vital events—including reporting cause of death and providing access to legal proof of registration—for all individuals by 2030”.

The WBG has been collaborating with development partners to strengthen the CRVS systems of LMICs through lending operations, technical assistance projects, advisory services and analytics, and sharing of knowledge and lessons learned at various international, regional, and national conferences and fora and through publications. The WBG employs a multisectoral approach and has three initiatives that contribute to improving CRVS:

i. Strategic Actions Program for Addressing Development Data Gaps: This program supports improving the quality and quantity of country-level data for monitoring the SDGs.
ii. Identification for Development (ID4D) initiative: This is a multisectoral initiative across the WBG on improving digital identification systems.
iii. Global Financing Facility (GFF): This activity is aimed at improving the health of women, children, and adolescents, and supports the strengthening of CRVS systems.

In addition, the WBG, in collaboration with several organizations, has developed a comprehensive CRVS eLearning course which provides practical tools and approaches to achieving twenty-first-century state-of-the-art CRVS systems that are linked to identity management systems and tailored to local contexts. This course covers various topics, such as institutional arrangements; legal framework; birth, death, marriage, and divorce registration; vital statistics; and identity management systems. The course is free and open to everyone; it has been completed by government CRVS teams, health professionals, and many others in more than 50 countries globally.

Based on these collective experiences and strategies that have been planned and implemented in improving CRVS systems in LMICs, the authors of this series have prepared eight manuscripts covering different aspects of CRVS.

### What articles are contained in this issue?

1. *Benefits of Linking Civil Registration and Vital Statistics with Identity Management Systems for Measuring and Achieving Sustainable Development Goal 3 Indicators*. Linking the civil registration and identity management systems is key to improving access of individuals to social services such as education, health, social welfare, and financial services. By assigning unique identification numbers at birth registration, and then using these numbers to link individuals’ data from civil registration, the identity management system, and other functional registers, including registers for migration and health care, individual beneficiaries can be identified more accurately for the efficient delivery of services. In addition, more accurate and disaggregated population values can be obtained to measure SDG 3 indicators. This paper discusses how this broader sharing of data across sectors can contribute to improving the effectiveness and efficiency of health service delivery, health coverage programs, and achievement of universal health coverage.
2. *A Multisectoral Institutional Arrangements Approach to Integrating Civil Registration*, *Vital Statistics and Identity Management Systems*. This paper reviews the essential components of a recommended institutional arrangements framework for an integrated CRVS and identity management system. Examples from countries that have successfully implemented the recommended components of an integrated CRVS and identity management system are presented.
3. *Unique Health Identifiers for Universal Health Coverage*. Achieving universal health coverage (UHC) is important; however, countries cannot implement effective and efficient UHC programs without being able to identify every member of the population to target some (e.g., the poor, children, and refugee populations, etc.) with essential and quality health services, and UHC progress cannot be adequately monitored without disaggregated data. This paper describes how civil registration linked to an identity management system facilitates the implementation and monitoring of UHC programs. Country examples are provided to demonstrate how UHC was achieved using unique health identifiers.
4. *Cloud-based Services for Electronic Civil Registration and Vital Statistics Systems*. This paper examines the hosting options for electronic CRVS systems, particularly the use of data centers versus cloud-based solutions. A data center is a facility that houses computer systems and associated hardware and software components, such as network and storage systems, power supplies, environment controls, and security devices. An alternative to using a data center is cloud-based hosting, which is a virtual data center hosted by a public cloud provider. The cloud is used on a pay-as-you-go basis and does not require purchasing and maintaining of hardware for data centers.
5. *eLearning course for Improving Civil Registration and Vital Statistics Systems*. This paper considers an approach to capacity building for improving CRVS systems, namely a CRVS e-Learning course developed collaboratively by global CRVS experts representing several organizations. The course aims to provide practical tools and approaches to building and maintaining state-of-the-art CRVS systems that are linked to identity management systems and are tailored to local contexts. Information on the design, delivery, and evaluation of the CRVS course thus far is presented, along with information on the challenges and benefits of the course’s preparation and delivery in three languages.
6. *A Missed Opportunity: Birth Registration Coverage is Lagging Behind Bacillus Calmette–Guérin (BCG) Immunization Coverage and Maternal Health Services Utilization in Low- and Lower-Middle-Income Countries*. The paper examined birth registration and immunization coverage rates in low- and middle-income countries using Demographic and Health Survey and Multiple Indicator Cluster Survey data from 2000 to 2017. National birth registration rates continue to fall behind childhood immunization rates. Majority of the countries with disparity between birth registration and BCG vaccination of more than 50 percent were from Sub-Saharan Africa. The gap between birth registration and immunization coverage in LMICs suggests that there is the potential for leveraging immunization and maternity care programs to increase birth registration rates.
7. *Economic Analysis of Producing Vital Statistics Using Civil Registration Data in Lao People's Democratic Republic*. The authors of this paper adapt a framework for economic analysis developed by Jimenez-Soto et al. to assess the cost-effectiveness of producing vital statistics from civil registration data, population survey, and population census. It showed that a complete and accurate CRVS system ranked highest, followed by the census, and a population survey. In addition to increasing cost-effectiveness for producing vital statistics, robust civil registration systems support improving the efficiency of public service delivery, leading to further cost savings for the country.
8. *Obstacles to Birth Registration in Niger*: *Estimates from a Recent Household Survey*. This paper analyzes the reasons for low birth registration in Niger and measures how solving various obstacles to registration faced by parents could help improve registration rates. Measures of potential gains in registration rates from different actions are provided, such as providing services closer to household residence, reducing out-of-pocket costs of registration, among others. Simulations are done of the potential effect on overall registration rates if various obstacles leading to low registration rates are resolved. The results can help inform policy options to increase birth registration.

### Factors that contribute to stronger CRVS systems

In addition to the lessons learned and recommendations presented in the eight manuscripts included in this series, there are other factors that contribute to stronger CRVS systems that the authors have observed from working with officials in several countries on improving these systems. First, it is very important to have a national coordinating committee (including ministries and development partners) with an anchor ministry/agency to oversee the development and implementation of a CRVS investment plan. Because CRVS involves several sectors dealing with different types of vital events, coordination is key to its success. Second, a comprehensive assessment of the CRVS system should be completed as one of the first steps to review the system’s current status and to identify weaknesses. Third, findings of the comprehensive assessment should feed into developing a costed investment case/strategic plan. In this process, prioritizing and sequencing of activities of the strategic plan is critical. From 2010, at least 30 countries in Africa have conducted CRVS assessments and developed plans in Africa [8], and 37 in Asia and the Pacific [9, 10]; the majority of them have established high-level coordination committees as well as technical working groups. The major challenges relate to implementation due to limited country capacity and lack of donor coordination. One of the priority areas is to improve the legal framework to support the proper functioning of national CRVS institutions. To aid this process, Bloomberg Philanthropies Data for Health Initiative has developed a tool that can be used to review a country’s legal framework for CRVS [11] and the United Nations Statistics Division is collaborating with several organizations to produce a *handbook on civil registration*, *vital statistics and identity management systems*: *communication for development*. Political commitment is essential to sustainability, and one of the ways of demonstrating national financial commitment to strengthening CRVS systems is to increase the share of public expenditures used to strengthen components of CRVS.

## Conclusion

Based on collective experiences and strategies that have been planned and implemented in strengthening CRVS systems in LMICs, the eight manuscripts in this series have been prepared, each covering different aspects of CRVS, to improve understanding of the importance of CRVS.

## Data Availability

Not applicable

## Abbreviations

CRVS: civil registration and vital statistics
GFF: Global Financing Facility
ID4D: Identification for Development
LMIC: low- and middle-income countries
SDG: Sustainable Development Goals
UHC: universal health coverage
WBG: World Bank Group
